# Items of General Interest

**Published:** 1916-01

**Authors:** 


### Items of General Interest.

Medical authorities are investigating the claims of Dr. A. D. Serrell Cooke and Dr. V. Gabriel, Paddington Infirmary, London, whose researches are said to have proved that garlic juice is a powerful antiseptic and, unlike others, harmless to human tissues.

The first case in which the remedy was used at Paddington Infirmary was that of a young girl, whose condition was precarious on account of the extreme affection of the right lung. When other means had failed, the infirmary physicians applied the garlic remedy. In two days the wound was normal. The patient is now rapidly recovering.

So powerful are the powers of garlic juice that to rub a small quantity on the back of your hand it will be on your breath in a very few minutes, say the discoverers.

The treatment as used at Paddington Infirmary has been to wash out the wound with garlic juice twice daily. Most of the subjects at this institution have been patients from blood poisoning. In all cases the remedy has produced results leading to the absolute cure of the subject.

The *Lancet*, Great Britain's leading medical journal, discussing the discovery, suggests that it be speedily taken to England's battlefields where Red Cross nurses and members of the Royal Army medical corps may administer it to wounded soldiers, hundreds of whom die simply because tetanus sets in before they can be removed to base hospitals for proper treatment.

---

Two monkeys at Johns Hopkins hospital are being fitted with eye glasses that will entail a severe strain on the eyes, causing imperfect vision and in other ways confuse the recording nerve of the eye-ball. This is being done in order to ascertain just what part the thyroid gland plays in diseases that are supposed to develop because of a lack on the part of this gland to perform its proper function.

---

Dr. James Hogan, of San Francisco, asserts that a solution of gelatine in water injected into the circulatory system will restore the blood pressure in cases where excessive losses of blood have exhausted the patient. Injections of this solution, he says, are quite as effective and successful as the transfusion of blood from the veins of a strong healthy person. He has gone to Europe, where he will use his discovery in the war hospitals.

A. and M. College is to have a new $50,000 hospital within the next year.

---

Great interest has been aroused in Berlin medical circles by the notice that Dr. Kraus of Buenos Aires has discovered a serum for the cure of whooping cough. In spite of the fact that little is known of the inventor in bacteriological circles, it is said that tests of the serum assure its success.

---

December 12th was "Tuberculosis Sunday" in Beaumont. All the pulpits in the city were filled by physicians, who spoke on the prevention of tuberculosis. Last year more than 100,000 churches and other organizations helped to spread the gospel of good health in the United States.

---

Corpus Christi has inaugurated a commendable and far-reaching reform in organizing a Charity Dispensary, where the poor and suffering may have the services of the city's best physicians absolutely free of charge. Two of the leading drug stores have agreed to furnish the unfortunate poor with drugs at cost. This is a humane movement that should be adopted by every city in Texas.

---

New York has recently opened a Radium Sanitarium for the treatment of cancer and other malignant diseases. The Sanitarium has 300 milligrams of the mineral, which is valued at $36,000. The Sanitarium will be conducted along the lines of the Radium Institute of London.

---

One of the largest enterprises undertaken in Kerrville lately is the establishing of a modern sanatorium, which will be known as the Mountain Park Sanatorium. The main building is made of concrete and contains thirty-four rooms, wide galleries, a roof garden, a modern heating plant. Mr. W. H. Chambers, the business manager, has purchased eight hundred acres, which will be laid out in parks and driveways. The Sanatorium will have its own private herd of Jersey cows and its own poultry yards. Everything is being done to make the establishment up to date in every particular.

---

Dr. Manuel Perez Amador, director of the government institute for biological research, has announced the discovery of a method for the taking of X-ray photographs without the use of radium, Crookes' tubes or expensive apparatus of any kind. Employing an ordinary plate holder of a camera, Dr. Amador placed the body of a lizard on the outer surface of the opaque side under which was a photographic plate. A short distance above the opaque slide he suspended an ordinary tin shade of an electric lamp, to the inner surface of which crystals of highly purified white sulphur had been made to adhere. The resultant radioactive rays penetrated the body of the lizard and the opaque slide and imprinted a perfect image of the skeleton and the internal organs of the reptile on the plate inside the holder. A large number of scientific men witnessed the demonstration.

Dr. Amador claims to have discovered a cheap substitute for radium during his experiments of the past eight years with the radio-active properties of white sulphur.

---

At a luncheon given by Dr. M. L. Graves, Chairman of the local Committee of Arrangements for the next meeting of the State Medical Association, it was definitely decided to hold the meeting on May 9, 10 and 11. The luncheon was held at the Galvez Hotel, and was attended by the President, Dr. Moody, the Secretary, Dr. Holman Taylor, and Dr. Jno. T. Moore, of Houston, and a number of other prominent officials.

---

The Sleeper-Davis Memorial Hospital at Pekin, China, was dedicated on the fifth of November. The new hospital is a five-story structure erected by the Methodist Episcopal Church of the United States at a cost of $18,000. It has accommodations for 150 patients, is heated by steam, lighted by electricity, equipped throughout with modern sanitary plumbing, and provided with diet kitchens capable of preparing both Chinese and foreign foods for the patients. All patients entering the hospital will be examined by American doctors, who have Chinese assistants.

---

Dr. Bloodgood of the Johns Hopkins University declared, in a talk before the Southern Medical Association, that the question of eradicating of cancer is simply a question of teaching individuals the cause of the trouble. The proper treatment of the common fever blister, he mentioned as one of the precautions necessary in such cases.

---

Negroes of Tulsa, Oklahoma, are financing a hospital for the exclusive use of their race. Although not barred from the regular hospitals, facilities for treating negro cases are limited, and for this reason wealthy members of the race started a movement for an adequate and up-to-date hospital for their own use.

---

The American board of commissioners for foreign missions has announced that they need fifteen surgeons and physicians in mission fields. Nine are needed in China, four in Turkey, one in Africa, and one for relief work in Serbia.

---

The East Dallas Medical Society met in regular session in Mesquite, and elected the following officers for the coming year: Dr. J. M. Armstrong, President; Dr. Ridgell, Vice-President; Dr. J. B. Knight, Secretary.

---

Alfred Jennings, a cattleman of near Ellsworth, Kansas, is ill with a disease that has been diagnosed as foot and mouth disease by Dr. S. J. Crumbine, Secretary of the Kansas State Board of Health.

---

The new Wichita General Hospital opened for business and has already had a number of patients. A number of operations have also been performed by the local physicians, and the institution is meeting its requirements handsomely.

---

E. W. Donnelly, self-styled "the ranger doctor of San Saba," was convicted of practicing medicine without a license. He claimed that he did not use medicines, and that there was no one who was able to give him examinations for a license to practice. He was fined $50 and one day's imprisonment.

---

The joint city and county hospital, recently completed at Wichita Falls, is now open for patients. The building, which is one of the first in Texas to be erected under the joint hospital law passed in 1913, was erected on $50,000 in bond issues, half being voted by the city and half by the county. The building proper cost about $40,000, and is modeled closely after larger institutions in Kansas City and other places.

---

Quinine has advanced from 25 cents an ounce to $2.50 in the past eighteen months, resulting in profits to speculators of $500,000 in the United States and $1,500,000 abroad. Brokers are believed to have accumulated 200,000 ounces, or almost a year's supply. Coal tar derivatives used as a substitute for quinine have advanced 400 per cent. Glycerine has advanced from 21 cents a pound before the war to 55 to 60 cents, while Norwegian oil has advanced from $19 a 30-gallon barrel to $85. Whatever of heroism or sacrifice of humanity there may be about war, dries up and shrivels to zero in the mind of the speculator who sees a chance to make excessive profits of someone's necessity. The moral difference between the crooked poker player and the man who speculates in food supplies or medicines is this: The poker player is profiting from his foolish adversary's love of excitement and risk, and willingness to "take a chance"; the speculator is profiting from the hunger or the sickness of those who must at any price have what he holds. It's a great sport, going after 1000 per cent; but sometimes it doesn't look quite so good.

---

Dr. W. S. Leathers, Dean of the Medical Department of the University of Mississippi, is the new Chairman of the Southern Medical Association Health Department. Dr. L. B. McBryor, of North Carolina, is the new Secretary.

